# Femtosecond Laser-Assisted Donor and Recipient Preparation for Bowman Layer Transplantation

**DOI:** 10.3390/jcm14124362

**Published:** 2025-06-19

**Authors:** Rosemarie Schlosser, Annekatrin Rickmann, Peter Szurman, Silke Wahl, Berthold Seitz, Philip Wakili, Lisa Julia Müller, Philipp Ken Roberts, Karl Thomas Boden

**Affiliations:** 1 Eye Clinic Sulzbach, Knappschaft Hospital Saar, 66280 Sulzbach, Germany; 2 Department of Ophthalmology, Saarland University Medical Center, 66421 Homburg/Saar, Germany

**Keywords:** Femtosecond Laser (FSL), Bowman layer transplantation, keratoconus, femtosecond laser Ziemer LDV Z8, low-energy femtosecond laser

## Abstract

**Objectives**: A Ziemer LDV Z8 femtosecond laser (FSL) was used to obtain optimal cutting parameters with precise settings for donor and recipient preparations for Bowman layer transplantation. **Methods**: Of 48 human research corneas examined, 32 were used for Bowman layer preparation (donor) and 16 for pocket preparation (recipient) using the LDV Z8 FSL. The cutting thickness of the Bowman layer, pocket depth, and corresponding laser settings were varied. The quality of sections was evaluated based on the occurrence of adhesions, bridges, or perforations. Histological specimens were prepared and analyzed. **Results**: Preparation of the Bowman layer and recipient pocket was possible using all selected settings. The thinner the Bowman layer and the more superficial the pocket preparation, the higher the risk of perforation was. Considering the fact that the Bowman layer was cut as thinly as possible, a Bowman layer thickness of 30 µm showed a 100% success rate. Bowman layers cut at 25 µm had a lower success rate (50%). The pocket depth of 150 µm showed a 100% success rate in the preparation. Histological processing revealed smooth, precisely cut edges of Bowman layers and pockets. Implantation into the pocket was successful in all cases. **Conclusions**: Both Bowman layer and pocket preparation were technically and surgically feasible using the LDV Z8 FSL, and the prepared Bowman layers were thinner than those reported in previous studies. The optimal Bowman layer thickness was 30 µm, and a resection depth of 150 µm was used to prepare the pockets safely.

## 1. Introduction

Keratoconus, which mainly afflicts young patients, is a bilaterally progressive non-inflammatory disease that presents as corneal stromal thinning with conical deformation of the cornea accompanied by biomechanical weakening [1]. In advanced stages of the disease, severe visual impairment occurs due to high myopia, irregular astigmatism, and corneal scarring [1]. In 12–20% of cases, the disease may necessitate corneal transplantation [2] using either penetrating keratoplasty (PKP) or deep anterior lamellar keratoplasty (DALK) [3]. With a thickness of approximately 8–12 µm, the Bowman layer is a corneal layer consisting of tightly interwoven collagen fibrils that significantly contribute to the stability of the cornea [4]. The pathomechanism of keratoconus is hypothesized to involve the destabilization of the Bowman layer, which leads to disease progression and thus, corneal ectasia [5]. Less invasive methods aim to transplant an isolated Bowman layer into the middle stroma to improve long-term corneal stability, achieve corneal flattening, and prevent the progression of ectasia with a corresponding loss of visual acuity [6], thereby avoiding the need for PKP or DALK, as well as their associated intraoperative and postoperative complications [7]. In Bowman layer transplantation (BLT), a pocket is created in the stroma of the recipient, into which an isolated donor Bowman layer is placed as an inlay [8]. The Bowman layer has been mainly prepared manually [9]. However, this manual preparation is quite complex and demanding, and may lead to loss of the transplant. Therefore, we aimed to test whether the Bowman layer could be prepared using the femtosecond laser (FSL) Ziemer LDV Z8, and determined the optimal cutting parameters for both the Bowman layer and pocket, attempting to cut the Bowman layer as thinly as possible and the pocket as superficially as possible, where the latter was positioned as closely as possible to the physiological location of the Bowman layer.

## 2. Materials and Methods

### 2.1. Study Design

**Figure 1:**
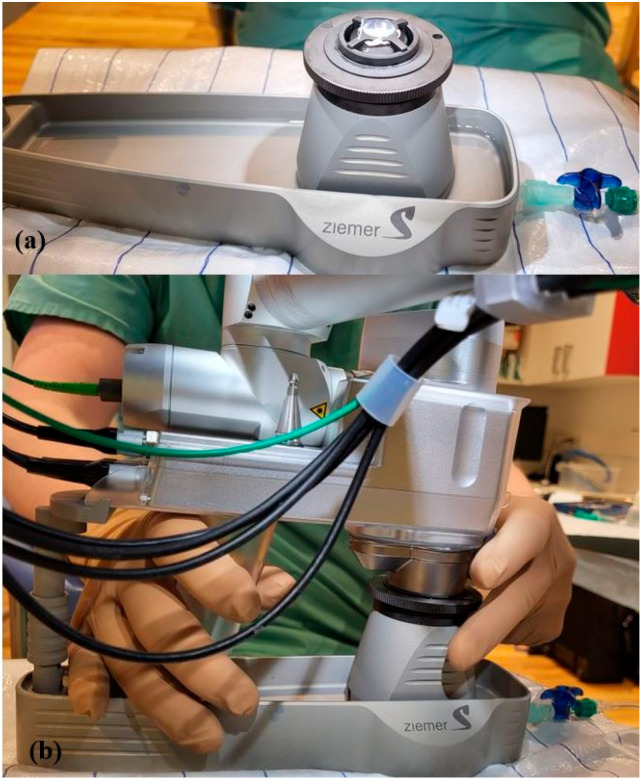
Experimental setup: (**a**) the cornea clamped in the artificial anterior chamber (**b**) with subsequent docking of the second handpiece to the laser arm.

**Figure 2:**
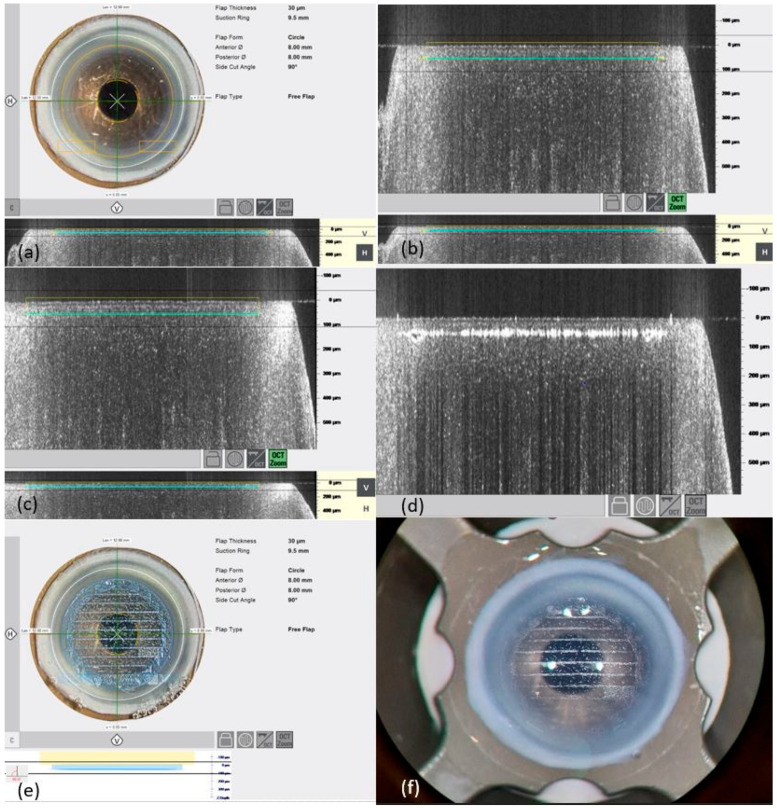
Illustration of Bowman layer preparation by creating a “free flap cut”. (**a**) Representation of the Bowman layer immediately before cutting. (**b**) Representation of the OCT with the planned settings prior to cutting, showing the horizontal cut. (**c**) Representation of the OCT with the planned settings prior to cutting, showing the vertical cut. (**d**) Illustration of the OCT after sectioning the Bowman layer. (**e**) Graphic user interface after performing sectioning by the laser. (**f**) Live photo of the top view of the donor cornea, showing the scatter of the artificial anterior chamber after cutting the Bowman layer using the femtosecond laser.

Human corneas were obtained from the German Society for Tissue Transplantation (DGFG) with the written consent of the donors’ relatives for research. This study complied with the principles of the Declaration of Helsinki and was approved by the local ethics committee (Saarland Medical Association, No. 07/17). A total of 48 human corneas (*n* = 48) were prepared with the femtosecond laser (Ziemer LDV Z8, Port, Switzerland). Of these, 32 corneoscleral slices were used for the preparation of Bowman layers; the remaining 16 slices were used for the preparation of recipient corneal pockets for the Bowman layers of the donors. **Tissue preparation:** The corneoscleral rim was placed epithelial-side-up on an artificial anterior chamber (Ziemer, Port, Switzerland). First, manual abrasion of the epithelium was performed. Then, the flexible handpiece of the FSL (Ziemer LDV Z8, Port, Switzerland) was docked onto the anterior chamber, and the second part of the handpiece attached to the laser arm was connected to the first part of the handpiece (Figure 1). The applanation interface was used for this purpose. All further steps are displayed on the graphical user interface (GUI) (Figure 2). Optical coherence tomography (OCT) integrated with the LDV Z8 allowed the positioning of the graft to be adjusted and control of the layer’s thickness (Figure 2).

**Figure 3:**
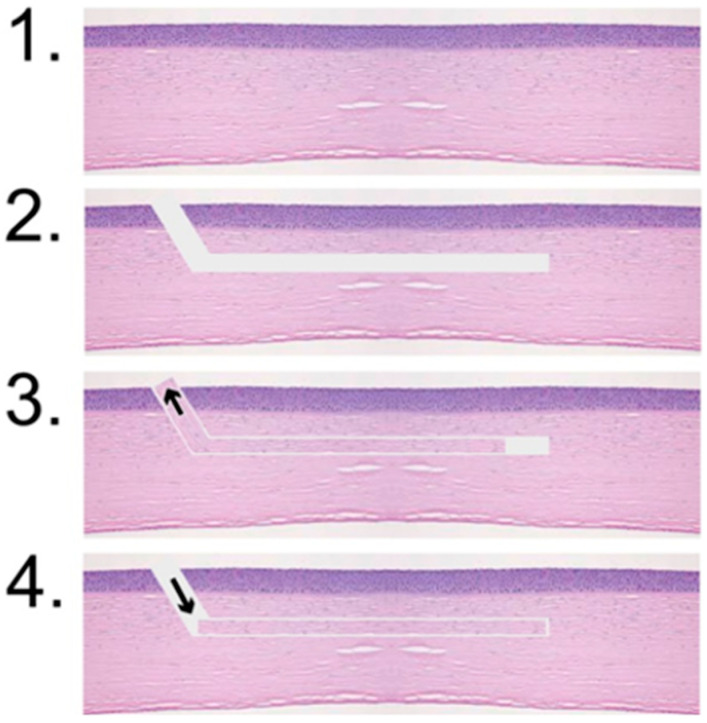
Schematic representation of the surgical procedure: 1. cornea of the recipient; 2. preparation of the pocket in the recipient cornea (recipient); 3. preparation of the Bowman layer of a different cornea (donor cornea); and 4. implantation of the donor Bowman layer into the recipient cornea in which the pocket was prepared.

The trephination thickness of the donor Bowman layer was set to the thinnest possible option, and the depth of the recipient pocket oriented to the physiological position of the Bowman layer was as shallow as possible without leaving an uncut area. These parameters were based on previous studies conducted by Van Dijk et al., who performed these procedures using the LDV Z6 [7]. The successfully prepared Bowman layers were inserted into the corresponding corneal pockets, as schematically shown in Figure 3. A Bowman layer preparation was considered successful if it was present as a free flap directly after the laser procedure or if adhesions could be loosened and no perforation occurred.

### 2.2. Donor Bowman Layer

Using the FSL, a total of 32 Bowman layers with five different thicknesses were prepared. Among these 32 Bowman layers, thicknesses of 45 µm (*n* = 12), 40 µm (*n* = 8), 35 µm (*n* = 4), 30 µm (*n* = 4), and 25 µm (*n* = 4) were analyzed. At the beginning of this study, it was not entirely clear to what extent the technical requirements would allow the preparation of different lamella thicknesses, so a larger quantity of thicker lamellae was initially prepared. In the course of this study, no more human corneas were available to prepare the same number of samples for each thickness group. In general, the safety settings of the laser platform had to be modified to prepare the Bowman layer at the time of implementation, which was performed in consultation with and in the presence of a representative as well as an engineer from Ziemer, as no settings were prespecified for the preparation of such a Bowman layer at the time. The modified parameters were based on the standard settings of the basic program of the LDV Z8 FSL for Bowman layer preparation; Table 1 lists these parameters.

As a test, for the final 45 µm Bowman layer preparation the energy used to make the side cut was reduced from 85% to 68%, and the cutting speed was changed from 40 mm/s to 50 mm/s. In a 25 µm thick Bowman layer, the speed of the stromal cuts was minimally increased (from 14 mm/s to 14.5 mm/s), and the applied energy for the stromal cut was slightly reduced (from 75% to 70%) on a trial basis.

### 2.3. Recipient Pockets

Out of a total of 16 pockets, 5 were prepared with a resection depth of 300 µm, 7 with a resection depth of 250 µm, 2 with a resection depth of 150 µm, and 2 with a resection depth of 100 µm. At the beginning of this study, reference was made to the previously published data of Melles et al. for pocket preparation, which corresponded to a dissection plane for pocket preparation of approx. 50% of the stromal depth. Later, a more superficial dissection was performed. Similar to the lamellar preparation, only a limited number of human corneas were available for this study for ethical reasons, resulting in an unequal distribution of the number of samples in the groups. The corresponding settings are shown in Table 2.

### 2.4. Histology

Histological processing was performed to visualize the cut edges. After preparation, specimens were fixed in 4% formaldehyde, embedded in paraffin, and cut into sections. Individual sections were stained with hematoxylin and eosin (HE) or periodic acid–Schiff (PAS) according to standard procedures [10]. Images were obtained using a BX60 microscope (Olympus, Hamburg, Germany) and the imaging software cellSens Dimension 1.11 (Olympus).

## 3. Results

### 3.1. Preparation of Bowman Layers

Of the 32 Bowman layers, 12 were prepared with a thickness of 45 µm, whereby 6 Bowman layers were prepared without complications. A further 2 of the 12 Bowman layers were successfully dissected after the adhesions were loosened. Due to a collapsed anterior chamber or perforations when releasing the adhesions and bridges, 4 of the 12 Bowman layers had to be discarded.

Furthermore, 8 of the 32 Bowman layers were prepared with a thickness of 40 µm. As the first two of four Bowman layers showed perforations, and a further two of four Bowman layers showed adhesions and bridges that could be successfully removed, a further three Bowman layers were successfully prepared after changing the laser settings (by reducing the energy for the stromal cut from 80% to 75% and reducing the energy for the lateral cut from 85% to 80%). Under these optimized settings, posterior perforation only occurred in one Bowman layer.

Another 4 of the 32 Bowman layers were prepared with a thickness of 35 µm, with the energy for the marginal cut set at 80% and for the stromal cut at 75%. Four out of four Bowman layers were prepared with smooth and precise cutting edges and used for implant placement. A “free flap” situation without adhesions or bridges was found in three of four Bowman layers.

While maintaining these settings, in four of four Bowman layers with a 30 µm thickness, a “free flap” situation without adhesions or bridges was achieved.

In the first of four Bowman layers with a thickness of 25 µm, a gas ingress occurred in the center, as well as multiple adhesions, so that a setting change was made. By changing the energy for the stromal cut from 75% to 70% and increasing the speed for the stromal cut from 14 to 14.5 mm/s, two of the four Bowman layers were prepared with the formation of adhesions that could be loosened. Posterior perforation occurred in another Bowman layer in which the energy of the lateral incision was reduced from 80% to 75% as a test.

### 3.2. Preparation of Recipient Pockets

Out of the 16 corneal pockets prepared using the FSL, 5 were prepared with a 300 µm resection depth. Of these, four were successfully prepared and used for implantation of the donor Bowman layer. Due to posterior perforation, one out of five had to be discarded. For all seven pockets with a resection depth of 250 µm, preparation was successful. After changing both the energy as well the speed for the incision and stromal cut, one of the two pockets was cut at a resection depth of 150 µm with adhesions that could be released. The energy for the incision cut was increased from 110% to 130%, the energy for the stromal cut was reduced from 100% to 80%, and the speed of the incision cut was increased from 15 mm/s to 40 mm/s, while the speed for the stromal cut was increased from 9 mm/s to 13 mm/s. The second pocket was prepared without adhesions or bridges.

In pocket preparation, of two pockets with a resection depth of 100 µm, a further reduction in the energy for the stromal cut from 80% to 75% and an increase in the speed of the stromal cut from 13 to 14 mm/s, while maintaining the other settings for the incision cuts, resulted in perforation in one pocket, while the second pocket was prepared without complications.

### 3.3. Histology

**Figure 4:**
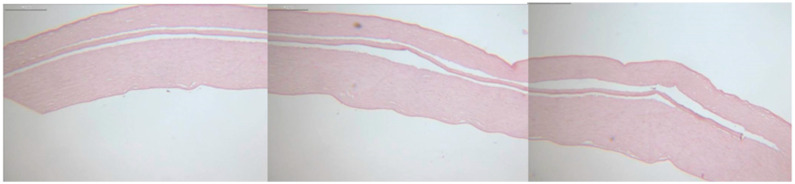
HE staining assembly showing a transplanted Bowman layer in one of the prepared corneal pockets at 10× magnification (BX60 microscope; Olympus, Hamburg, Germany). Scale bar = 200 µm.

Histological analysis revealed smooth, precisely cut edges of the Bowman layers and pockets, as shown in Figure 4. Due to the shrinkage artifacts caused by kerosene embedding and the subsequent staining process, no exact correlation could be established between the values set during preparation and the histopathological sections.

## 4. Discussion

To date, BLT has been used to strengthen and flatten thin corneas resulting from advanced keratoconus [11] and for the treatment of subepithelial opacities after photorefractive keratectomy [11]. In particular, patients with advanced progressive keratoconus are eligible for this procedure if they have good contact lens correction but are increasingly intolerant to contact lenses and cannot undergo collagen crosslinking because of their pachymetry values [8]. Previously, in such cases, PKP or DALK were the favored therapeutic procedures [2]. In contrast to PKP, DALK has the distinct advantage of not disrupting the endothelium.

The main difficulty of BLT lies in its intraoperative risk, both in the preparation of the recipient pocket and the donor Bowman layer. Specifically, during manual preparation of the donor Bowman layer, a rupture or perforation can occur, or the Bowman layer may be prepared with the incorrect thickness [12]. In all these cases, the donor Bowman layer grafts must be discarded [8]. Luceri et al. examined corneal density and higher-order aberrations (HOAs) after BLT and determined that the incidence of HOAs, like spherical aberrations, decreased for both anterior and posterior corneal surfaces, while the prevalence of corneal backscattering increased [6].

To optimize the visually determined smooth surface, it seems reasonable to use an FSL. Using a low-energy FSL with short light pulses (10^−15^ s), tissue can be cut gently and precisely by photodisrupting the tissue using a wavelength of 1053 nm, causing minimal tissue damage [9]. Our histological workup affirmed this, as smooth surfaces were created for both donor and recipient preparation. By contrast, Parker et al. showed that the Bowman layer preparations of the FSL group were thicker overall (mean of 37 µm), but had a relatively smooth posteriorly cut edge, whereas the manually cut preparations showed only the Bowman layer with irregular remnants of dispersed stroma [7]. However, the thickness of the Bowman layer prepared using the FSL varied in their study [7]. Compared with previous studies carried out by Melles et al. [7], who used the LDV Z6 model, we employed the LDV Z8. This later model has the advantage of integrating OCT, enabling precise visualization throughout the process. In addition, the Z8 model differs from the Z6 model in terms of precision, as the Z8 is also used in refractive surgery for lenticule preparation. Therefore, the feasibility limits of the newer model were tested here for the first time. We demonstrated that a 30 µm thin Bowman layer could be consistently prepared with little risk of perforation or formation of adhesions while choosing the optimum laser settings, referring to the theory that the lower the speed used and the smoother the cuts, the fewer bridges and adhesions that need to be manually removed afterwards. The risk of perforation can be reduced accordingly. In this study, the best energy level was determined to guarantee that the dissection in the tissue had smooth cut edges while reducing gas penetration and avoiding unnecessary damage to the surrounding tissue. These Bowman layers were thinner than those prepared in previous studies [7], signifying a change in BLT regarding approaching the physiological Bowman layer thickness, which is 10−20 µm.

Recipient pockets with varying resection depths were also prepared using the FSL, as in previous studies [7]. In our test series, it was both technically and surgically possible to prepare pockets with a resection depth of up to 100 µm; however, the risk of perforation was lower at 150 µm than at 100 µm, making the pockets less shallow than the ones previously prepared [7]. However, only a very small number of pocket preparations were performed.

Physiologically, the Bowman layer lies directly under the corneal epithelium [13]. It is questionable whether a deeper implantation into the stroma or an approximation of the physiologic position achieves advantages in terms of postoperative astigmatism, visual rehabilitation, and stability. Deeper implantation would possibly lead to more stability, but at the same time to a higher immune response [14]. Postoperative increases in backscattering-inducing glare and lower contrast sensitivity have been described after BLT, which could be reduced using a more superficial pocket preparation [6]. In addition, the extent to which the thickness of the lamellae affects visual acuity, astigmatism, and stability needs to be investigated in further studies.

Compared to manual preparation, the FSL ensures both precise planning and execution. The use of the FSL also provides advantages in terms of customization and faster visual rehabilitation. Thanks to integrated optical coherence tomography (OCT) and the corresponding visualization of the graphic user interface, visualization is always possible, which leads to increased intraoperative safety during the surgical procedure [15]. Despite the safe cutting of the Bowman layer with the FSL, surgical skill is relevant, as surgeon-induced perforation can also occur. Limitations in the use of the femtosecond laser are found in corneal scars and in highly irregular pachymetry [5].

Histological imaging of the Bowman layers and pockets showed flat, unrolled Bowman layers with clear-cut edges, and the Bowman layers were inserted into the pockets without complications. The safety and reproducibility offered by the FSL allow a larger group of users to perform this procedure successfully, as the preparation is less reliant on appropriate corneal benches or manual surgical experience. However, a company representative must be present to adjust the settings appropriately, as the platform’s security settings must be lowered. The transferability of individual Ziemer platform data to other platforms remains to be investigated.

By the time this study was performed in 2023, BLT for both donors and recipients had been performed for the first time using the LDV Z8 FSL. With a total of 32 prepared Bowman layers and 16 pockets, this was the first series of experiments to test whether Bowman transplantation is feasible using the Z8 model in human corneas.

In conclusion, corneal Bowman layer and pocket preparation is not only feasible using the Z8, but a Bowman layer with a thickness of 30 µm can be prepared precisely and safely under permanent monitoring by the integrated OCT. Thus, we demonstrated for the first time that thinner Bowman layers can be prepared using the Z8 compared with those prepared using the Z6 [7]. In principle, Bowman layers with thicknesses below 30 µm can also be prepared using the FSL, but with an increase in the risk of perforation. Pockets of varying depths can also be prepared; the most superficial pocket in our study was 100 µm. Due to the low perforation rate, pocket preparation was found to be safer, after this series of tests, at a minimum depth of 150 µm.

## Figures and Tables

**Table 1:** Inferred laser settings for donor Bowman lamella preparation.

Thickness of Bowman layer (µm)	30
Diameter of suction ring (mm)	9.5
Anterior diamter (mm)	8
Posterior diamter (mm)	8
Angle of side cut (°)	90
Velocity of side cut (mm/s)	40
Energy of side cut (%)	80
Velocity of stromal cut (mm/s)	14
Energy of stromal cut (%)	75
Posterior surface (µm)	5

**Table 2:** Inferred laser settings for recipient pockets.

Resection depth (µm)	150
Incision diamter (mm)	9.5
Width of tunnel (mm)	5
Pocket diameter (mm)	9
Angle of side cut (°)	30
Input position (°)	sup
Energy of incision cut (%)	130
Velocity of incision cut (%)	40
Energy of stromal cut (%)	80
Velocity of stromal cut (mm/s)	13

## Data Availability

The data are not publicly available due to privacy restrictions. The data that support the findings of this study are available from the corresponding author upon reasonable request.
